# Cas9‐PF, an early flowering and visual selection marker system, enhances the frequency of editing event occurrence and expedites the isolation of genome‐edited and transgene‐free plants

**DOI:** 10.1111/pbi.13118

**Published:** 2019-05-01

**Authors:** Yong Liu, Jianmin Zeng, Cheng Yuan, Yushuang Guo, Haiqin Yu, Yongping Li, Changjun Huang

**Affiliations:** ^1^ Yunnan Academy of Tobacco Agricultural Sciences Key Laboratory of Tobacco Biotechnological Breeding National Tobacco Genetic Engineering Research Center Kunming China; ^2^ Key Laboratory of Molecular Genetics Guizhou Institute of Tobacco Science Guiyang Guizhou China

**Keywords:** CRISPR‐Cas9, early flowering, visual selectable marker, genome editing, tobacco

Dear editor,

CRISPR/Cas9‐mediated gene editing technology has been successfully and widely used in plants (Ma *et al*., 2016). Currently, more often application of CRISPR/Cas9 entails the stable integration of the Cas9 endonuclease and single‐guide RNA (sgRNA) genes into plant host genomes (Yin *et al*., 2017). Hence, selecting T0 plants which carry active transgenes would theoretically enhance the frequency of editing event occurrence. Moreover, assessment of heritability and phenotypic stability in genome‐edited plants requires elimination of the Cas9/sgRNA T‐DNA cassette in the T1 generation (He *et al*., 2018; Lu *et al*., 2017; Mao *et al*., 2018). Therefore, efforts to reduce generation time (from T0 plants to T1 seeds) and efficiently screen transgene‐free plants with an edited genome in the T1 generation will have a major impact on genome editing technology for both basic research and crop improvement.

The *Flowering Locus T* (*FT*) has been shown to encode a mobile signalling molecule that may function as a major component of florigen, involved in the triggering of early flowering in plants (Putterill and Varkonyi‐Gasic, 2016). Therefore, we speculated that expression of FT might reduce the time required for a complete transgenic breeding cycle and thus accelerate the generation of genome‐edited and transgene‐free plants.

The *PRODUCTION OF ANTHOCYANIN PIGMENTS 1* (*PAP1*) gene controls the accumulation of anthocyanins in *Arabidopsis thaliana* (Borevitz *et al*., 2000). Several lines of evidence demonstrate that overexpression of PAP1 results in distinctively purple‐coloured leaves in different plants (Zhang *et al*., 2014). Theoretically, when a *PAP1* expression element is incorporated into a CRISPR/Cas9 vector, the activity and presence of the T‐DNA cassette in transgenic plants could be monitored based on the purple leaf phenotype. This could be utilized as a phenotype‐based marker for genome‐edited T0 plants and subsequent selection of transgene‐free T1 plants.

A PF cassette containing tandem PAP1 and NtFT expression elements was designed and synthesized by GenScript (Nanjing, China). Since Cas9 and sgRNA were expressed from 35S and U3 promoters, respectively, the Cestrum yellow leaf curling virus (CmYLCV) promoter and the Arabidopsis ubiquitin 10 (AtUbi10) promoter were chosen for PAP1 and NtFT expression, respectively, to prevent the use of duplicate promoters (Figure 1a). The synthesized PF cassette was directly inserted into the *Hind*III site of the pRGEB31 (Xie and Yang, 2013) CRISPR/Cas9 vector (Cas9) to construct a new CRISPR/Cas9 vector, pRGEB31‐PF (Cas9‐PF, Figure 1a), using the ClonExpress II One Step Cloning Kit (Vazyme, Nanjing, China). To compare the efficiency of targeted mutagenesis and the effectiveness of transgene‐free T1 plant screening, the original Cas9 vector and our modified Cas9‐PF vector were armed with gRNAs targeting the first exon of the tobacco (*Nicotiana tabacum* cv. Yun87) *Eukaryotic Translation Initiation Factor 4E* (*EIF4E1.S*) gene (Figure 1a), a recessive resistance gene to *Potato virus Y* (PVY) in tobacco (Julio *et al*., 2015).

**Figure 1:**
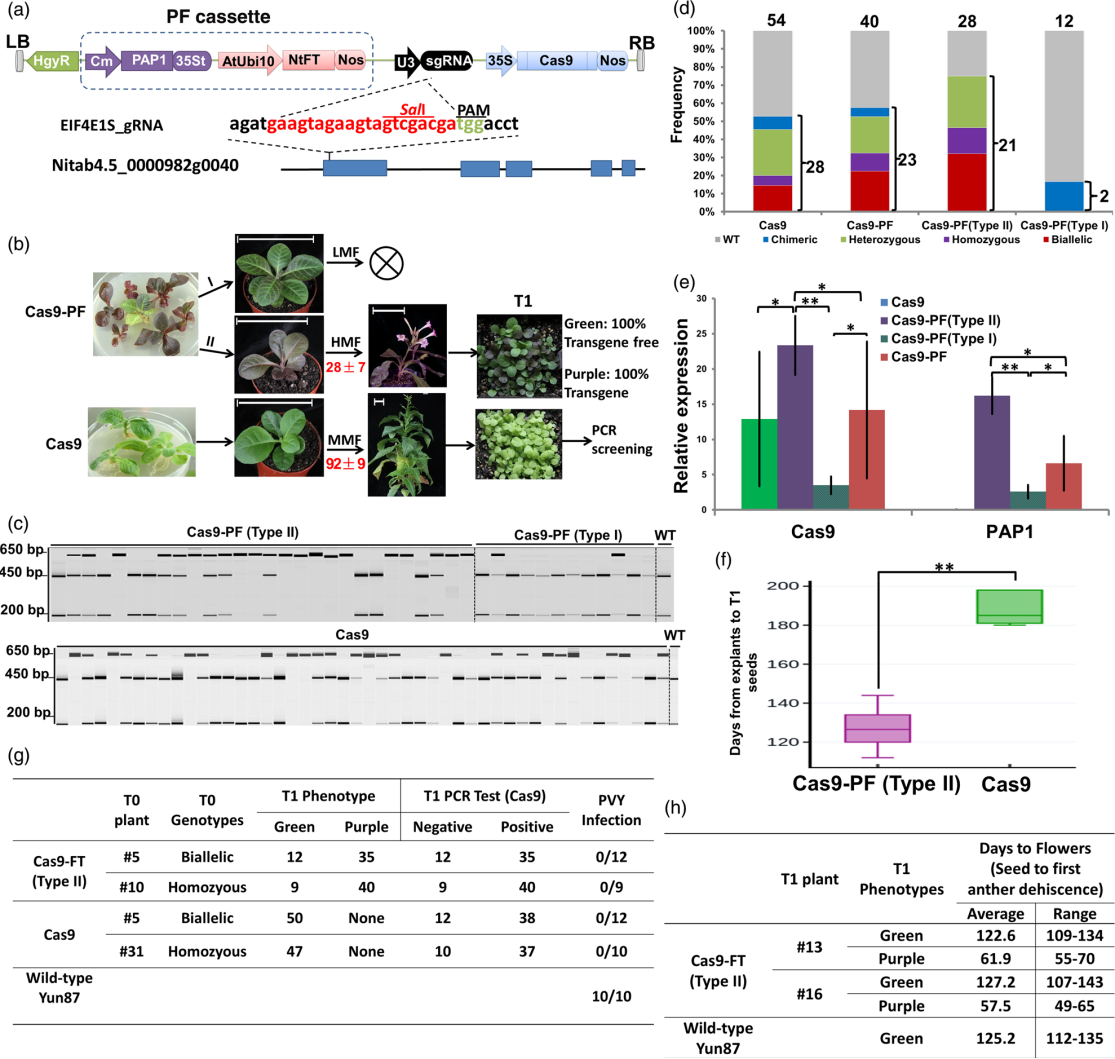
Improving the frequency of editing event occurrence and accelerating transgene‐free genome‐edited plant isolation using a Cas9‐PF toolset. (a) Schematic diagram of the PAP1 and FT (PF cassette) co‐expression CRISPR/Cas9 system, Cas9‐PF. (b) Workflow chart for the rapid isolation and visual screening of stably inherited, genome‐edited, transgene‐free mutants in tobacco using the PF cassette. Red numbers represent days from six‐leaf seedlings to flowering. LMF, low mutant frequency; MMF, moderate mutant frequency; HMF, high mutant frequency. Bar = 10 cm. (c) PCR/RE assay to detect Cas9/gRNA‐induced *EIF4E1.S* mutations of T0 transgenic plants transformed with Cas9‐PF or Cas9 constructs. PCR products were digested by *Sal*I. The presence of the *Sal*I resistance band indicates that the *EIF4E1.S* target has been edited. WT, wild‐type. (d) Histogram showing the frequency of WT, chimeras, biallelic, homozygous and heterozygous mutants in T0 populations transformed with Cas9 or Cas9‐PF constructs. The numbers above each column indicate total number of plants used for analysis, while those on the right‐hand side represent the number of mutants. (e) Relative expression of Cas9 and PAP1 in Cas9, Cas9‐PF, Cas9‐PF (Type I), and Cas9‐PF (Type II) T0 plants determined by qRT‐PCR. (f) Box plot diagram representing a time consumption comparison of conventional Cas9 to Cas9‐PF (Type II) during one whole transgene generation. (g) Chart demonstrating that the all green seedlings from the T0 plants lacked transgene elements. In addition, all transgene‐free T1 plants homozygous or biallelic for the *EIF4E1.S* mutation were inoculated with and found to be resistant to PVY (biological test results > 9) at 14 days postinoculation (dpi). (h) Chart showing that transgene‐free progeny plants revert to the normal phenotype of flowering time (biological test results ≥ 10). * denotes a significant difference at *P* < 0.05 while ** indicates a significant difference at *P* < 0.01, based on a two‐tailed Student's *t*‐test.

In contrast to the normal green leaves observed in all T0 transgenic seedlings with the Cas9 vector, T0 transgenic seedlings containing the PF expression cassette were classified into two types based on the intensity of the purple colour (Figure 1b). The leaves of type I seedlings were a mosaic blend of light purple and green, while type II seedlings developed pure dark purple leaves (Figure 1b). To determine whether PAP1 ectopic expression alters CRISPR/Cas9 efficiency per se, the overall mutation frequencies in the target site were calculated by polymerase chain reaction (PCR) amplification, restriction enzyme digests (PCR‐RE, Figure 1c) and Sanger sequencing. As expected, a similar mutation frequency was observed in T0 populations transformed with PAP1‐coexpressed CRISPR/Cas9 systems compared to those without PAP1 (57.5% vs 51.9%) (Figure 1c,d). However, we found that type I plants had an extremely low mutation frequency (16.7%), with chimeric mutations, while the mutation frequency dramatically increased for type II plants (75%), demonstrating a strong link between the gene editing efficiency of CRISPR/Cas9 and the intensity of the PAP1‐induced leaf colour phenotype (Figure 1c,d). To investigate this correlation, quantitative real‐time PCR (qRT‐PCR) analysis was performed to determine the abundance of Cas9 and PAP1 transcripts in T0 plants. The results showed that type II plants accumulated dramatically higher levels of both Cas9 and PAP1 transcripts than type I plants (Figure 1e). Thus, the expression of PAP1 can be utilized as a selective marker to increased propensity to contain genome editing events in T0 generation transgenic plants.

To validate our hypothesis that co‐expression of FT in plants can reduce generation time and decrease the total time required to complete a transgenic breeding cycle, 10 homozygous or biallelic *EIF4E1.S* gene‐edited T0 lines derived from type II plants or Cas9 vector transgenic plants were transplanted from the medium into soil and further evaluated in a climate‐controlled growth chamber. Under 14 h light/10 h dark, plants carrying AtUbi10:FT flowered after approximately 28 days; however, no bolting or flowering occurred in plants without FT until about 92 days (Figure 1b). Overall, plants with FT completed a transgenic breeding cycle (from explant preparation to T1 seeds) in approximately 127 days, which was significantly shorter than the range for transgenic plants without FT (180–198 days) (Figure 1f).

Approximately 50 T1 plants from each single homozygous or bi‐allelic *EIF4E1.S* gene‐edited transgenic T0 tobacco line were grown to assess whether the design of our system simplified the selection of transgene‐free T1 plants. In contrast to the all green phenotypes observed in the progeny of T0 transgenic plants with the pRGEB31‐derived vector, the T1 segregants of type II T0 transgenic plants had either purple or green phenotypes (Figure 1b). To detect whether the transgenes were present in the T1 plants and to confirm the accuracy of the visible colour selection based on anthocyanin pigmentation, DNA was extracted from each plant and tested using *Cas9* primers. As shown in Figure 1g, all green T1 progeny from type II T0 transgenic plants did not contain the transgene elements, demonstrating that our visible selection strategy was highly effective for identifying transgene‐free T1 plants. (Figure 1g).

Plantlets of the transgene‐free T1 plants containing a homozygous or biallelic *EIF4E1.S* mutation resulting in premature stop codons were then evaluated for PVY resistance. All wild‐type plants were found to be susceptible. In contrast, all of the tested T1 plants containing a homozygous or biallelic *EIF4E1.S* mutation were found to be resistant to PVY. Taken together, we have demonstrated for the first time the utility of CRISPR/Cas9 technology for the generation of tobacco plants with genetic resistance to PVY (Figure 1g). In addition, no undesirable epigenetic effects on flowering time and leaf pigment were observed once PF cassette was segregated away in T1 transgene‐free progeny plants (Figure 1h).

The results of this proof‐of‐concept study clearly demonstrated that our PAP1 and FT co‐expression system can accelerate the generation and identification of target gene‐edited and transgene‐free plants within a single, short generation, with significant improvements in the frequency of editing event occurrences using CRISPR/Cas9. Our technology greatly reduced time and labour, with almost no cost associated with isolating transgene‐free, genome‐edited plants. Another advantage of our strategy is that PAP1 and FT, as well as their orthologs, have conserved roles in anthocyanin accumulation and early flowering, respectively, in both monocot and dicot species. (Putterill and Varkonyi‐Gasic, 2016; Zhang *et al*., 2014). Therefore, our strategy can be easily adopted for other plant species that can be transformed through tissue culture or floral dipping. Our strategy will be even more beneficial for crops that have long life cycles.

## Conflict of interest

The authors declare no conflict of interest.
